# Visual event boundaries trigger forgetting despite active maintenance in visual working memory

**DOI:** 10.1167/jov.24.9.9

**Published:** 2024-09-11

**Authors:** Joan Danielle K. Ongchoco, Yaoda Xu

**Affiliations:** 1 Department of Psychology, Yale University, New Haven, CT, USA; 2 Department of Psychology, University of British Columbia, Vancouver, BC, Canada

**Keywords:** visual working memory, event segmentation, real-world animations

## Abstract

The contents of visual perception are inherently dynamic—just as we experience objects in space, so too events in time. The boundaries between these events have downstream consequences. For example, memory for incidentally encountered items is impaired when walking through a doorway, perhaps because event boundaries serve as cues to clear obsolete information from previous events. Although this kind of “memory flushing” can be adaptive, work on visual working memory (VWM) has focused on the opposite function of *active maintenance* in the face of distraction. How do these two cognitive operations interact? In this study, observers watched animations in which they walked through three-dimensionally rendered rooms with picture frames on the walls. Within the frames, observers either saw images that they had to remember (“encoding”) or recalled images they had seen in the immediately preceding frame (“test”). Half of the time, a doorway was crossed during the delay between encoding and test. Across experiments, there was a consistent memory decrement for the first image encoded in the doorway compared to the no-doorway condition while equating time elapsed, distance traveled, and distractibility of the doorway. This decrement despite top–down VWM efforts highlights the power of event boundaries to structure what and when we forget.

## Introduction

The building blocks of perception involve not just objects in space but also events in time: breakfast, followed by a shower, followed by one's morning commute. And just what counts as an event? Previous work has found that event boundaries arise out of a wide range of cues, ranging from more explicit cues, such as the introduction of a new character or goal in a film (Huff, Meitz, & Papenmeier, 2014) to subtler ones, such as a change in the implicit statistics in our immediate environments (Schapiro, Turk-Browne, Norman, & Botvinick, 2015). These event representations can arise out of entirely visual cues, such as flashes of color (Tauzin, 2015), changes in motion trajectories (Zacks, 2004), or sudden motion offsets (Ongchoco & Scholl, 2019). A great deal of work has explored what gives rise to these event representations because they *matter*—especially insofar as they serve as the underlying units over which perceptual and cognitive processes operate.

### “Memory flushing” at event boundaries

Perhaps the most well-explored effect of event boundaries on cognition is on memory. In particular, memory seems to be a function not just of continuous time elapsed but also of the number of events experienced. This may be best appreciated in the phenomenon in which walking through doorways seems to impair memory (Radvansky & Copeland, 2006; for a review, see Radvansky, 2012). In these experiments, subjects are typically asked to pick up different objects (e.g., a blue cone) while walking through different rooms. During their walk, they would sometimes be probed for their memory of the features of the objects they encountered (e.g., the color of the cone). Their ability to recall the features of the objects was impaired when they crossed a doorway during their walk compared to when they did not, equating time and distance traveled. This effect has been demonstrated for object features and words (Wang, Ongchoco, & Scholl, 2023) and is robust across memory tasks, whether recognition vs. recall (Pettijohn & Radvansky, 2018a; Seel, Easton, McGregor, Buckley, & Eacott, 2018) and visual properties, from room size, wall opacity, to visual distinctiveness (Logie & Donaldson, 2021; Pettijohn & Radvansky, 2015; Radvansky, Krawietz, & Tamplin, 2011). This effect occurs for active navigation (e.g., crossing doorways in virtual reality) but also works in entirely visual animations, as when one is just watching oneself walk through a doorway (Pettijohn & Radvansky, 2018b; Wang et al., 2023). It also does not seem to reflect mere context change, as these effects have been observed even when spatial layouts of the rooms are entirely identical pre- and post-door (Wang et al., 2023), and they still occur even when people return to the room (Radvansky, 2012).

This phenomenon of forgetting at event boundaries has been explained in various ways in the literature—mostly as a function of “updating” of event models (Kurby & Zacks, 2008; Radvansky, 2012), perceptual interference, or attentional lapses at the boundary (Güler, Adıgüzel, Uysal, & Günseli, 2023). But, more recently, this phenomenon has also been discussed in terms of a metaphor of “memory flushing” (Ongchoco & Scholl, 2019; Wang et al., 2023). When the statistics of our immediate environments have dramatically changed, as when one moves from one room to another, it might be helpful to “flush” or clear information (not necessarily in an all-or-none manner) that no longer applies to the new event—similar to how one might empty a cache or buffer in a computer program. Thus, although we typically think of forgetting as a limitation, it may be adaptive for memories to be reset or refreshed at key moments (Davis & Zhong, 2017; Richards & Frankland, 2017). Event boundaries such as doorways may serve as the cues that the statistics of the world are about to change and, in turn, trigger forgetting.

The function of memory, however, is not just for storing (and clearing) information that is encountered over time (e.g., objects in a room) but also for maintaining information in the face of incoming distraction and interference. How does active maintenance in visual working memory (VWM) interact with forgetting at event boundaries?

### Active maintenance in visual working memory

As we move through the world, we often need to store visual information that is no longer in view for brief periods of time. This VWM enables us to form a coherent and continuous representation of the external world, often interrupted by factors such as object occlusion and saccadic eye movements (Chen, Töllner, Müller, & Conci, 2018, Irwin & Andrews, 1996). Through top–down control and active maintenance, VWM also makes visual information available to support goal-directed behavior and thoughts (for a review, see D'Esposito & Postle, 2015). For example, cues to remember particular items can lead to benefits in memory retrieval later on (Souza, Rerko, & Oberauer, 2014; Williams & Woodman, 2010). To measure VWM performance, observers are typically presented with a set of target items that they are explicitly asked to remember (e.g., simple visual features or objects), followed by a blank delay, after which they are then asked to detect a feature change in the target items in a change detection paradigm (Luck & Vogel, 1997; Pashler, 1988) or report the feature of a particular target item in a continuous report paradigm (Zhang & Luck, 2008).

In everyday vision, however, we rarely see a blank screen while holding information in VWM; rather, the visual system is constantly bombarded with a continuous influx of visual information. A key challenge for VWM, then, is how to maintain information amidst distraction. When distractors are inserted during the delay period to mimic the real-world visual experience, although a small drop in memory performance has been noted in some studies, the bulk of information is still retained in VWM, indicating the ability of VWM to resist distraction (for reviews, see Lorenc, Mallett, & Lewis-Peacock, 2021; Xu, 2017). Distractor resilience can be achieved with distractor suppression or blocking through frontal mechanisms (Lorenc et al., 2021). Targets and distractors can also form independent and orthogonal representations during VWM in the human posterior parietal cortex, a key area involved in VWM maintenance (Xu, under review). This can accommodate the simultaneous representations of both targets and distractors while at the same time minimizing interference.

### The current study: Memory flushing versus active maintenance

VWM seems to be resistant to distractions encountered between encoding and test, so how can we understand this in the context of event boundaries that might be crossed during the same delay period? Here we pit these two cognitive operations of memory flushing on the one hand and active maintenance on another. Can active maintenance overcome memory flushing at event boundaries, or is there a component of such forgetting that cannot be avoided? To test for maintenance in the face of incoming information, we presented items serially (as opposed to simultaneously), allowing us to probe whether and which information gets degraded at event boundaries, despite being actively maintained in VWM. Being able to probe memory for each item can also elucidate the mechanism of memory flushing at event boundaries. If true to its name, then we should see some impairment for all items; otherwise, the impact of event boundaries on memory may be more nuanced than the metaphor implies.

We explored our question in the context of a VWM paradigm with an extended delay period (Bettencourt & Xu, 2016; Harrison & Tong, 2009). Observers watched themselves “walk” through three-dimensionally (3D) rendered rooms with picture frames on the walls in which observers either were presented with target images to remember (i.e., the “encoding” phase) or were asked to report which of an image pair they had seen in an immediately preceding frame in the room (i.e., the “test” phase). As with previous “walking through doorways” effects, the observers walked through a doorway between encoding and test phases for half of the time, while in the other half they did not. Critically, target images were presented serially during encoding so that observers could attend to each image individually, allowing us to assess which information will get impacted the most: Are all items in VWM impacted equally, or do event boundaries impact some more than others (e.g., the information encountered earlier versus later in the event)? Across Experiments 1 and 2, we tested for memory impairment at event boundaries while varying working memory loads, encoding times, and working memory content. In Experiment 3, we disentangled encoding and test order to further isolate the impact of event boundaries on some information over others.

## Experiment 1: Boundary-driven “forgetting” of visual working memory

We first replicated the original “walking through doorways” effect (Radvansky & Copeland, 2006) with a visual working memory task. In a continuous virtual museum “walk,” observers saw five images appear one after another in an “encoding” picture frame, “walked” to another picture frame (the delay period), and then reported which images they saw in a subsequent two-alternative, forced-choice (2AFC) task at “test.” Critically, half of the time, observers crossed an event boundary between encoding and test, whereas in the other half they did not.

### Method

#### Participants

One-hundred non-excluded observers (*M*_age_ = 27.58; 57% female, 40% male, 3% other) from the United States were recruited using the Prolific online platform. This sample size was determined from pilot testing and was fixed to be identical across the experiments reported here. All experimental methods and procedures were approved by the Yale University Institutional Review Board, and all observers confirmed that they had read and understood a consent form outlining their risks, benefits, compensation, and confidentiality and that they agreed to participate in the experiment.

#### Apparatus

After agreeing to participate, observers were redirected to a website where stimulus presentation and data collection were controlled via custom software written using a combination of HTML, CSS, JavaScript, PHP, and JsPsych libraries (de Leeuw, Gilbert, & Luchterhandt, 2023). Observers completed the experiment in fullscreen mode on either a laptop or desktop computer. (Because the experiment was rendered on observers’ web browsers, viewing distance, screen size, and display resolutions could vary dramatically, so we report stimulus dimensions below using pixel values.)

#### Stimuli

**Figure 1.**
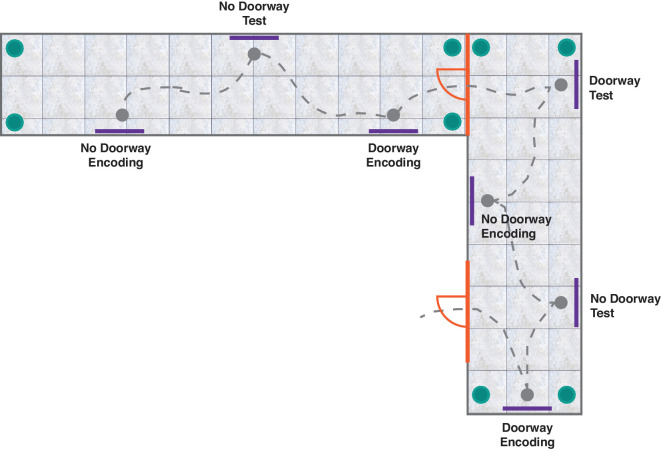
Sample floor layout of the video animation. At “encoding” picture frames, observers saw images appear one after another. At “test” picture frames, observers completed 2AFC trials to report which image they had seen in the immediately previous picture frame. Doorway trials (with the doorway indicated by the orange marks) and no-doorway trials alternated. The gray dashed line represents a caricature of the “path traveled” by the observer as the camera panned around the rooms, with gray discs representing stopping points throughout the animation. Purple lines along the walls represent picture frames. Green discs in the room corners represent furniture added to the room (e.g., plants or lamps). There were three viewing positions at the first room (at the start of the experiment) and four viewing positions for the rest of the other rooms (with doorway trials corresponding to first and last viewing positions in the room, and no-doorway trials corresponding to the middle viewing positions in the room).

The image dataset used for the VWM task contained 10 images of bicycles, 10 images of hangers, and 10 images of couches (taken from Jeong & Xu, 2017; Xu & Jeong, 2015). Image widths were 30% of the screen width of the browser. Each observer was presented with a video animation that simulated navigation in a virtual environment. Video animations were created using Sweet Home 3D. Each video animation featured 10 “rooms” through which the observer navigated. Each room had plants or lamps in the corners (indicated as teal circles in Figure 1) and had picture frames on the walls (indicated as purple lines along the walls in Figure 1), on which images would later be displayed. The picture frames were always on opposing walls of the room, and the opposing walls were always of different colors. The picture frames were arranged such that they could either have a doorway in between them or not. The animation always began with the no-doorway condition, where picture frames were not separated by any doorway, followed by a doorway condition, where picture frames were separated by a doorway, and so on. An animation had a total of 16 stopping points, for a total of eight trials.

**Figure 2.**
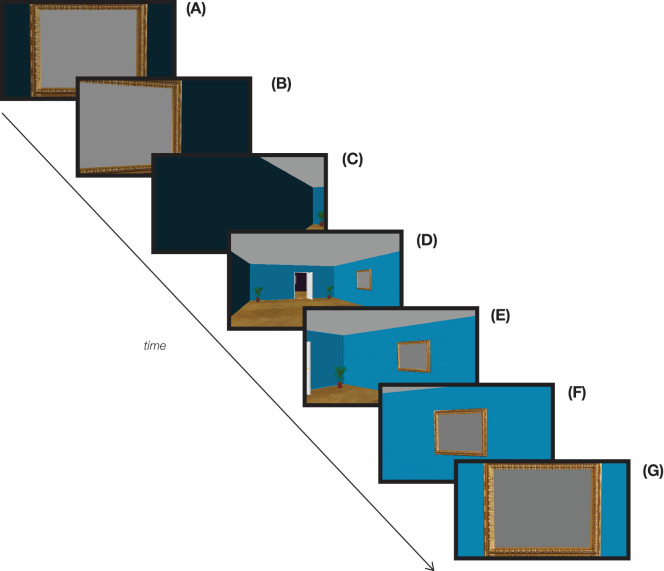
Sample frames from the video animation. (**A**) The camera faces the “encoding” picture frame on which images appear one after another. (**B**–**F**) The camera pans around the room to face the next picture frame. (**G**) The camera faces the “test” picture frame, on which a pair of images appear one after another, and observers report which image they saw in the immediately previous picture frame.

To ensure that any effects would not be a function of the particular rooms, three unique video animations were generated with different colored walls and floors. The animation of the “virtual walk” was created by using the camera and “Create a 3D video” function of the software. The path of the camera was set by identifying key positions where the camera would pass by during the 3D simulation. This path started with the camera view facing one of the picture frames (Figure 2A). The camera would then pan as if the observer were turning (Figure 2B) and moving around the room (Figures 2C to 2F), toward the next picture frame (Figure 2G). The viewing of picture frames was always separated by 12 seconds.^1^ Animations were rendered within Sweet Home 3D into 1280 × 720-pixel videos at 25 frames per second. These files were then converted into .mp4 files to increase web browser compatibility. Video animations were presented at 94% of the screen width of the browser.

#### Procedure and design

**Figure 3.**
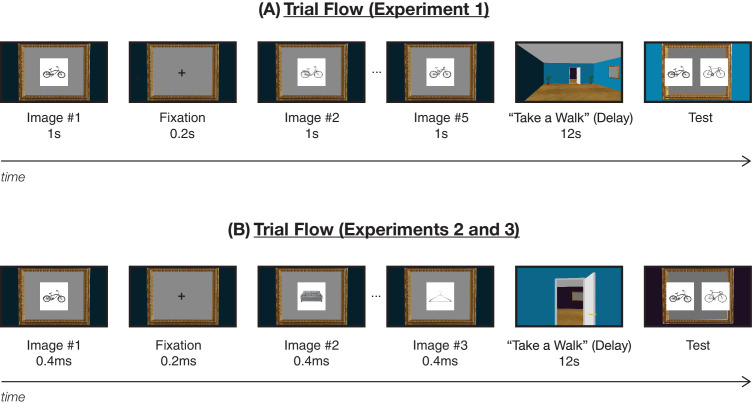
Trial flow. (**A**) Trial flow in Experiment 1, where five images appeared one after another for 1 second each, with a 0.2-second fixation cross in between. The encoding phase was followed by a 12-second delay during which the observers “took a walk,” after which the test phase began. (**B**) Trial flow in Experiments 2 and 3, where three images appeared one after another for 0.4 seconds each, with a 0.2-second fixation cross in between.

Each observer was presented with the video animation, and the instructions were overlaid on top of the first frame of the video animation. The animation then began playing continuously, pausing at picture frames in the room. In “encoding” picture frames, observers saw five images from the bicycle, hanger, or couch image sets appear one after another for 1000 ms each, with a 200-ms fixation cross in between image presentations. This encoding phase was followed by a 12-second delay period, during which observers “walked” across the room toward the next picture frame in the room. In the immediately following “test” picture frames, observers then saw two images appear to the left and right of a central fixation cross—and they simply chose which image they had seen in the previous picture frame (within a 2200-ms response time window). For the full trial flow, see Figure 3A for reference. Observers completed this 2AFC task five times during the test phase for each of the five images they saw during encoding in the same order, such that the first pair of images included the first image the observer previously saw and a foil image from the same category set, the second pair of images included the second image the observer previously saw and a foil image from the same category set, and so on. To equate order effects across trials and conditions, we always tested images in this order (but we investigated potential order effects more directly in Experiment 3).

All observers watched two complete animations (with two of three video animations randomly selected per observer) where each animation was comprised of eight trials: 2 room conditions (doorway vs. no-doorway) × 4 repetitions. Trials were always comprised of an encoding and a test section for each observer, with a self-timed break between animations.

#### Exclusions

Observers were excluded based on the following exclusion criteria: (a) those who reported a subjective attention level below 75% (*n* = 16), and (b) those whose overall accuracy was below 50% (*n* = 2). These observers were not included in the final set of 100 observers included in the analysis. Missed trials (*M* = 4.18, *SD* = 7.36) and trials with response times greater than 2 *SD* from the grand population mean (*M* = 2.00, *SD* = 2.02) were not included in the analysis.

### Results

**Figure 4.**
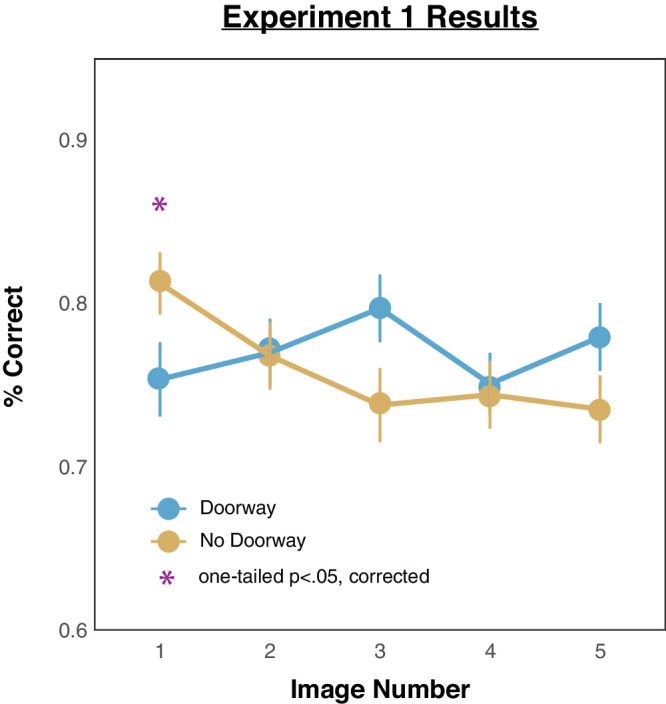
Results from Experiment 1. The *x*-axis depicts the order of the images that observers saw, and the *y*-axis depicts the average proportion correct from the 2AFC responses. Error bars depict standard errors of the mean.

We computed the percentages of correct responses across all the trials for all images. The resulting mean accuracy rates for each image position, broken down by doorway versus no-doorway, are depicted in Figure 4. Inspection of performance in the no-doorway condition (yellow line) reveals that the strongest memory was found for the first presented image above all the rest of the images. In contrast, inspection of performance in the doorway condition (blue line) reveals worse memory for the first presented image but not for the rest.

These initial impressions were confirmed with the following statistical analyses. A 2 (doorway vs. no-doorway) × 5 (image presentation positions) analysis of variance (ANOVA) showed that there was no main effect of room condition, *F*(1, 99) = 0.84, *p* = 0.363; there was a main effect of image presentation order, *F*(1, 99) = 4.24, *p* = 0.042; and there was a significant interaction between room condition and image presentation order, *F*(1, 99) = 9.50, *p* = 0.003. Based on previous work on “walking through doorways” effects, we predicted a directional effect between the doorway and no-doorway conditions. Pairwise one-tailed comparisons were thus conducted, and these revealed worse memory in the doorway condition than in the no-doorway condition for the first image, *t*(99) = 2.66, *p* = 0.023, *d* = 0.27, but not for any of the others: image 2, *t*(99) = 0.17, *p* = 0.431, *d* = 0.02; image 3, *t*(99) = 2.52, *p* = 0.017, *d* = 0.25; image 4, *t*(99) = 0.31, *p* = 0.474, *d* = 0.03; image 5, *t*(99) = 2.02, *p* = 0.038, *d* = 0.20. All *p* values reflect Benjamini–Hochberg corrected values (Benjamini & Hochberg, 1995).^2^

### Discussion

These results confirm the effect of walking through doorways on working memory, and they do not seem to be a function of the mere distractibility of doorways. To rule out this possibility, we computed the frame-by-frame image complexity of the video animations (reduced in resolution to account for textured walls) using a lossless compression algorithm (Ellis & Turk-Browne, 2019). This resulted in complexity values of 0.160 for the doorway animation and 0.175 for the no-doorway condition, where a lower proportion reflects greater redundancy and thus lower complexity. The results, therefore, could not be merely explained by greater visual complexity (and thus distractibility) driven by the doorway.

Moreover, these results demonstrate for the first time, to our knowledge, *what* information gets degraded at event boundaries. Whereas previous “walking through doorways” effects have always tested memory by having people remember either only one item at a time or multiple items simultaneously, the current paradigm revealed that event boundaries impact visual memories, especially memory for the first information that is encountered in an event.

## Experiment 2: Ruling out effects of load and item similarity

Results from the previous experiment suggested an effect of the event boundary on memory, but only for the first image that observers encountered. Why was memory impaired for this item and not the others? In particular, we noted that memories for the remaining items were all lower across conditions, which raised the possibility that working memory load was too high or the task was too difficult (Luck & Vogel, 1997; Xu & Chun, 2006) for us to observe the effect of the event boundary (i.e., perhaps we were seeing a floor effect)? Here, we reduced the load that observers had to remember (from five images to three), and we sampled images from different categories (as opposed to just one image set). We asked whether memory would still be impaired only for the first item, despite easing the working memory load.

### Method

This experiment was identical to Experiment 1 except where noted. One-hundred new non-excluded observers (*M*_age_ = 27.23; 62% female, 35% male, 3% other) participated (with this sample size chosen to exactly match that of Experiment 1). This time, observers had only three target images to remember (at 400 ms each), and each image was selected from a different category (bike, couch, or hanger), such that observers only saw one bike, one couch, or one hanger each time (Figure 3B). Observers were excluded based on the following exclusion criteria: (a) those who reported a subjective attention level below 75% (*n* = 17), and (b) those whose overall accuracy was below 50% (*n* = 0). Missed trials (*M* = 2.05, *SD* = 1.88) and trials with response times more than 2 *SD* from the grand population mean (*M* = 1.73, *SD* = 1.81) were not included in the analysis.

### Results

**Figure 5.**
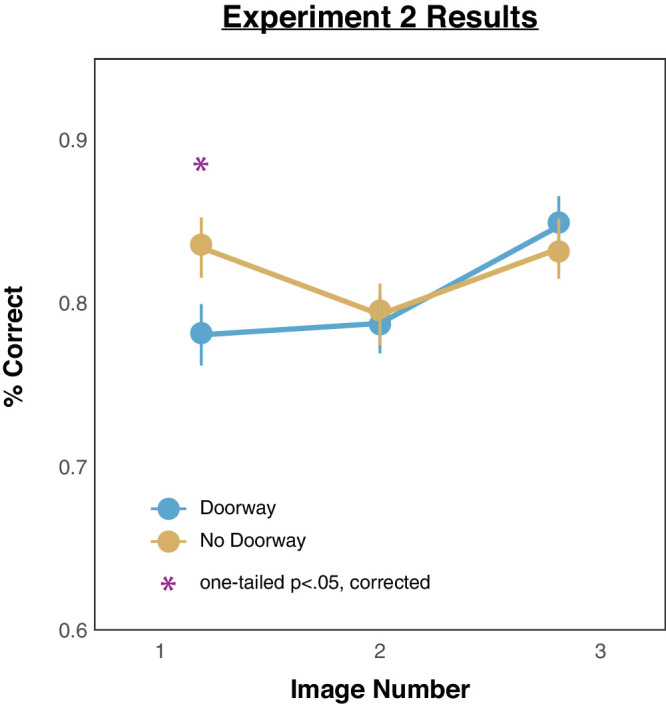
Results from Experiment 2. The *x*-axis depicts the order of the images that observers saw, and the *y*-axis depicts the average proportion correct from the 2AFC responses. Error bars depict standard errors of the mean.

We computed the percentages of correct responses across all the trials for all images. The resulting mean accuracy rates for each image position, broken down by doorway versus no-doorway, are depicted in Figure 5. Inspection of performance in the no-doorway condition (yellow line) reveals higher memory accuracy for the first and third presented images. In contrast, inspection of performance in the doorway condition (blue line) reveals worse memory for the first presented image but not for the other images. These initial impressions were again confirmed with the following statistical analyses. A 2 (doorway vs. no-doorway) × 3 (image presentation positions) ANOVA showed that there was no main effect of room condition, *F*(1, 99) = 1.22, *p* = 0.272; there was a main effect of image presentation order, *F*(1, 99) = 5.68, *p* = 0.019; and there was a significant interaction between room condition and image presentation order, *F*(1, 99) = 6.75, *p* = 0.011. Pairwise one-tailed comparisons (all *p* values reflect Benjamini–Hochberg corrected values) revealed worse memory in the doorway condition than in the no-doorway condition for the first image, *t*(99) = 2.58, *p* = 0.028, *d* = 0.26, but not for any of the other images: image 2, *t*(99) = 0.33, *p* = 0.617, *d* = 0.03; image 3, *t*(99) = 0.99, *p* = 0.406, *d* = 0.1.^3^

### Discussion

These results replicate those of the previous experiment, where memory impairment due to the event boundary (of the doorway) was again limited to only the first information encountered in the event. This was true even after reducing overall memory load and even after ruling out potential within-category interference (and the possibility that observers were just confusing different token images of the same type).

## Experiment 3: Probing the effect of image order

Thus far, we had consistently observed a decrement in working memory performance for the first images that observers were tested on. However, because observers were always tested with images in the same order as they were encoded, we cannot say whether the decrement was specific to the first image itself or just a function of which image was tested first. In this experiment, we replicated the previous “walking through doorways” effect in VWM, but this time reversing the order of the first two images tested.

### Method

This experiment was identical to Experiment 2 except where noted. One-hundred new non-excluded observers (*M*_age_ = 28.95; 34% female, 66% male, 0% other) participated (with this sample size chosen to exactly match those of Experiments 1 and 2). Observers saw three target images to remember, but, instead of being tested with images from categories in the same order they were encoded, we probed memory for the second image, followed by the first image, followed by the last image. Observers were excluded based on the following exclusion criteria: (a) those who reported a subjective attention level below 75% (*n* = 10), and (b) those whose overall accuracy was below 50% (*n* = 4). Missed trials (*M* = 1.81, *SD* = 1.50) and trials with response times more than 2 *SD* from the grand population mean (*M* = 1.96, *SD* = 5.22) were not included in the analysis.

### Results

**Figure 6.**
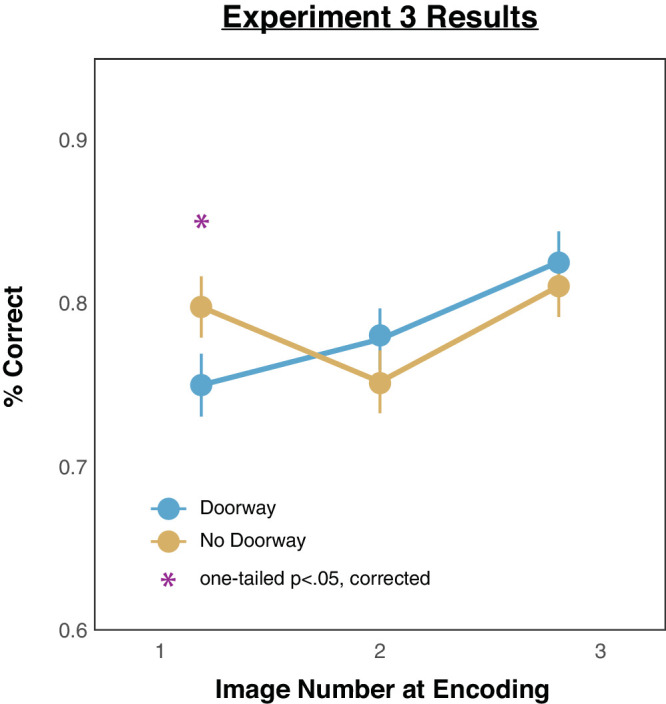
Results from Experiment 3. The *x*-axis depicts the order of the images that observers saw, and the *y*-axis depicts the average proportion correct from the 2AFC responses. Error bars depict standard errors of the mean.

We computed the percentages of correct responses across all the trials for all images. The resulting mean accuracy rates for each image position, broken down by doorway versus no-doorway, are depicted in Figure 6. Inspection of performance in the no-doorway condition (yellow line) reveals higher memory accuracy for the first and third presented images. In contrast, inspection of performance in the doorway condition (blue line) reveals worse memory for the first presented image but not for the other images. These initial impressions were again confirmed with the following statistical analyses. A 2 (doorway vs. no-doorway) × 3 (image presentation positions at encoding) ANOVA showed that there was no main effect of room condition, *F*(1, 99) = 0.04, *p* = 0.839; there was a main effect of image presentation order, *F*(1, 99) = 13.06, *p* < 0.001; and there was a significant interaction between room condition and image presentation order at encoding, *F*(1, 99) = 4.85, *p* = 0.030. Pairwise one-tailed comparisons (all *p* values reflect Benjamini–Hochberg corrected values) revealed worse memory in the doorway condition than in the no-doorway condition for the first image, *t*(99) = 2.4, *p* = 0.045, *d* = 0.24, but not for any of the other images: image 2, *t*(99) = 1.25, *p* = 0.267, *d* = 0.13; image 3, *t*(99) = 0.68, *p* = 0.415, *d* = 0.07.^4^

### Discussion

Here, even when we reversed the testing order of the first two images, we still replicated the effect from previous experiments that event boundaries can trigger memory impairment—in particular, reducing memory for the *first* presented images that observers encoded from the previous event. In other words, it is the order in which an image is encoded rather than the order in which an image is tested that determines the amount of forgetting after crossing a doorway.

## General discussion

One of the most familiar kinds of event boundaries that we cross everyday is a *doorway*, and the impact of “walking through doorways” on memory has been extensively explored in previous work. A challenge for VWM then is how to hold onto information in the face of these visual cues to forget. Here, we asked if active maintenance in VWM can overcome forgetting triggered by event boundaries. We did so by testing VWM for items that were sequentially encountered and that observers were actively maintaining during a VWM delay period while they either did or did not walk through a virtual doorway. This yielded two key results: (a) memory was only impaired for the first items (but not subsequent ones) that were encountered before walking through a doorway, and (b) this “walking through doorways” effect was not simply a function of VWM being taxed due to high load and within-item similarity. Thus, despite the active maintenance mechanism deployed to sustain items stored in VWM, memory loss due to crossing an event boundary was still present, indicating that this type of forgetting cannot be eliminated even when observers actively try to overcome it.

For any given event, we do not encounter all of the information from that event all at once at the beginning of the event, as has usually been the case in previous work. Instead, although some information can be immediately available at first glance when walking into a new room, moving through the room can reveal new information about different objects that may not have initially been in view or about objects with which we interact. Some information might be more relevant for behavior than others, and we may want to be able to hold onto it even across boundaries. Despite this mix of information that we accumulate within any event, whether or not all information gets impacted at event boundaries in preparation for a new event has not been addressed by prior studies, as all previous “walking through doorways” effects involved observers only remembering either one object at a time (Lawrence & Peterson, 2014; Radvansky & Copeland, 2006) or a set of words presented simultaneously (Wang et al., 2023). Thus, the current study has carved out the scope of “walking through doorways” effects by showing that information is not simply forgotten in an “all-or-none” fashion, and that some information is more prone to the sort of forgetting triggered by an event boundary.

It makes sense that information should not be equally forgotten when moving from one event to another, but why would the impact of event boundaries on memory be consistently seen for the first item that was encountered in a previous event and not for all the rest of the items? We speculate that the current results may reflect the existence of two types of VWM: one type that is more automatic (“by default”) and another type that is maintained in a more top–down fashion (i.e., through deliberate effort to sustain the representation). This distinction can be appreciated through a simple observation: Even without exerting effort to actively maintain visual information, we do spontaneously notice when an item is repeated. This has also been demonstrated neurally when the repetition of an item results in a lower functional magnetic resonance imaging (fMRI) response amplitude, also termed fMRI adaptation or repetition suppression (Grill-Spector, Henson, & Martin, 2006), even though observers are not trying to remember the item. Such an effect demonstrates the lingering neural trace left by an encoded item and can support later recall of the item. In the current study, the first items encountered in an event may benefit from such a neural trace similar to a sort of “primacy tag”, as what is typically found in serial recall in long-term memory (Murdock, 1962; Tan & Ward, 2000). This primacy for the first item in memory has also been found in other working memory studies (Siefke, Smith, & Sederberg, 2019), which similarly have found a primacy for the first items after background changes (such as changes in background color). A similar kind of tagging has been suggested by other work arguing for independent mechanisms of resetting and updating in working memory (Balaban, Drew, & Luria, 2018; Balaban & Luria, 2017). Final retrieval performance may thus reflect the interaction of these two processes across different forms of memory and should not be attributed solely to forgetting triggered by crossing a doorway.

Although such an automatic form of VWM may be independent of the sustained representation provided by top–down VWM control mechanisms, it appears to be vulnerable to the onset of event boundaries, perhaps because our visual system has evolved to automatically reprioritize resources in an optimal way in response to the demands of the new event. Our results show that crossing an event boundary may affect the automatic part of VWM but not the sustained part of VWM. Supporting this, we have also found that the priming effect in a speed judgment task is reduced by crossing a virtual doorway (Ongchoco et al., in preparation). Together, our results show that the doorway effect occurs at the perceptual level and may not be completely overcome even under top–down sustained attention and VWM efforts.

In addition to examining the doorway effect on VWM, the present study also tested VWM in a more ecological setting, where observers encountered information, navigated their environments, and then tried to retrieve or act on previously remembered information. This is an important direction for future research, as a core function of VWM—that perhaps even distinguishes it from other forms of memory—has always been for guiding online behavior. To act from one moment to the next, we need to be able to maintain information from moments just before. Indeed, VWM capacity has been related to measures of intelligence (Fukuda, Vogel, Mayr, & Awh, 2010) and has been identified as a core ability that guides and constrains how we process information as we move around the world. Probing VWM as we move through rooms and walk through doorways thus brings us a step closer to understanding how our memories shape and adapt to the ebb and flow of dynamic visual experience.

## Footnotes

1 Although this may seem like a long duration for a delay period relative to traditional behavioral VWM studies, this duration is not atypical for functional magnetic resonance imaging work (Bettencourt & Xu, 2016; Harrison & Tong, 2009). Moreover, previous work has suggested that VWM may be defined not simply by the length of the retention period but rather by whether active maintenance is sustained (Phillips & Christie, 1977).

2 When breaking trials down by the first versus second half of the experiment, we still observed the main interaction between room condition and image position at encoding, *F*(4, 392) = 4.51, *p* = 0.001, and there was no three-way interaction, *F*(4, 392) = 0.39, *p* = 0.811.

3 When breaking trials down by the first versus second half of the experiment, we still observed the main interaction between room condition and image position at encoding, *F*(2, 198) = 3.16, *p* = 0.045, and there was no three-way interaction, *F*(2, 198) = 1.98, *p* = 0.211.

4 When breaking trials down by the first versus second half of the experiment, we observed a marginally significant main interaction between room condition and image position at encoding, *F*(2, 198) = 2.96, *p* = 0.054, and a marginally significant three-way interaction, *F*(2, 198) = 2.79, *p* = 0.064.
